# Pseudoephedrine Induced Ischemic Colitis: A Case Report and Review of Literature

**DOI:** 10.1155/2018/8761314

**Published:** 2018-06-28

**Authors:** Muhammad Aziz, Asad Pervez, Rawish Fatima, Ajay Bansal

**Affiliations:** ^1^Department of Internal Medicine, University of Kansas Medical Center, Kansas City, KS, USA; ^2^Department of Gastroenterology and Hepatology, University of Kansas Medical Center, Kansas City, KS, USA; ^3^Department of Internal Medicine, Dow University of Health Sciences, Karachi, Pakistan

## Abstract

Ischemic colitis due to medications is common, and a number of cases have been described with pseudoephedrine as the culprit agent. We present here an interesting case of a healthy female with no risk factors who developed pseudoephedrine induced ischemic colitis. This case serves to remind the healthcare providers about the utmost importance of obtaining a comprehensive history to aid with the diagnosis.

## 1. Introduction

Ischemic colitis is caused due to the sudden interruption of blood supply of the bowel, which results in ischemic injury particularly to the watershed areas. Risk factors associated with ischemic colitis include age, female gender, cardiovascular conditions, autoimmune diseases, hypercoagulable state, and medications [1].

Various case reports have been published linking ischemic colitis to numerous medications [1]. We present an interesting case of a patient with no medical comorbidities who presented with ischemic colitis likely associated with pseudoephedrine.

## 2. Case Report

A 54-year-old previously healthy Caucasian female with otherwise unremarkable past medical history presented to emergency department with one-day history of hematochezia and abdominal pain. The patient described crampy left lower quadrant pain with no aggravating or relieving factors. She had a total of five bowel movements since symptom onset with the first bowel movement containing stool mixed with bright red blood followed by predominantly bloody stools. She took no medications on a regular basis and denied having a screening colonoscopy for colorectal cancer at age 50. She reported symptoms of upper respiratory tract infection (cold, sneeze, and cough) for which she took three doses of 120 mg pseudoephedrine purchased from a local grocery store for 1 day prior to symptom onset. Her maternal grandfather had prostate cancer but there was no significant gastrointestinal tumor history in the family. She was a nonsmoker and reported drinking socially (roughly one standard drink) once a week.

**Figure 1:**
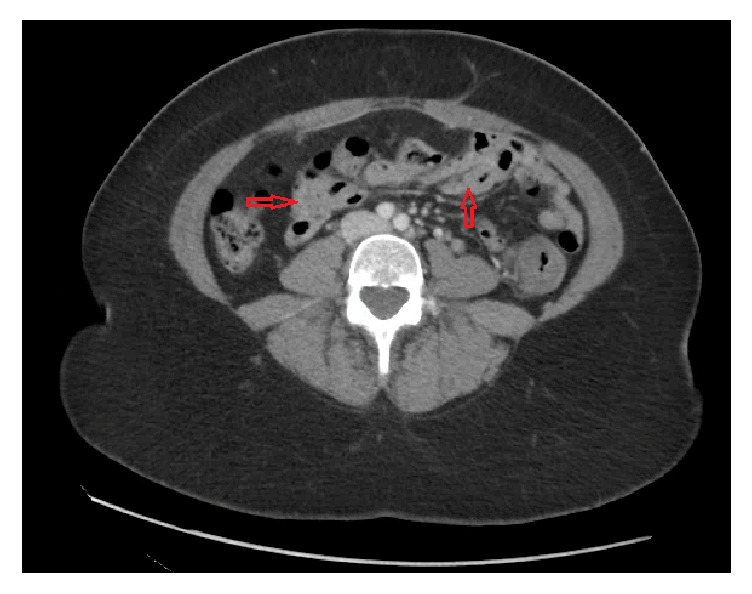
Transverse section of computed tomography scan demonstrating mild to moderate mural thickening of the descending/sigmoid colon (arrows) consistent with colitis without pericolonic abscess, ascites, or free air.

Her admission vitals were within normal limits. Physical examination was consistent with mild tenderness on the left side of abdomen and hypoactive bowel sounds. Rectal examination showed bright red blood without any stool in the rectal canal. Her laboratory values were significant for mild anemia with hemoglobin of 11.5 mg/dl, hematocrit of 34.5%, erythrocyte sedimentation rate 31 mm/hr, and C-reactive protein 2.15 mg/dl. A computed tomography scan revealed mild to moderate mural thickening of the descending/sigmoid colon consistent with colitis without pericolonic abscess, ascites, or free air (Figure 1). An infectious workup was obtained including blood cultures, stool cultures, gastrointestinal panel for* Clostridium difficile*, and gastrointestinal viruses but was negative. She was resuscitated with intravenous fluids.

**Figure 2:**
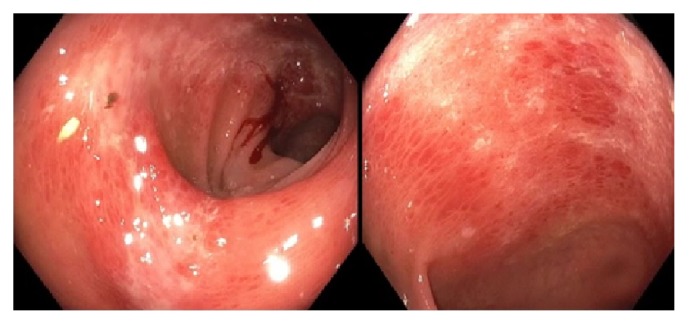
Colonoscopy demonstrating segmental moderate inflammation in the sigmoid colon, descending colon, and splenic flexure.

The patient underwent colonoscopy which demonstrated segmental moderate inflammation in the sigmoid colon, descending colon and splenic flexure along with internal and external hemorrhoids. There was evidence of submucosal hemorrhages with mild edema in the aforementioned segments of the colon (Figure 2). Endoscopic findings were highly suspicious of ischemic colitis. Several biopsies were obtained from the inflamed areas which exhibited focal lamina propria eosinophilic change with mild crypt attenuation and loss of goblet cells consistent with mild ischemic changes. There was no evidence of chronic inflammation.

She was observed in the hospital for 3 days and her diet was progressed slowly. Her bloody bowel movements ceased after 1 day in the hospital and patient was counseled and educated regarding avoidance of pseudoephedrine and over the counter medications for symptomatic management.

## 3. Discussion

This case demonstrates the occurrence of ischemic colitis in an otherwise healthy female who did not have any major risk factors that would predispose her to ischemic colitis. Medications are an increasingly common cause of ischemic colitis [1], which led us to do an extensive inquiry into over the counter drugs, herbal medications, complementary, and alternative management. The fact that the patient improved with conservative measures and has remained disease free since recovering from her initial injury favors medication as the culprit for causing ischemic colitis as opposed to a thrombotic or autoimmune disease. The patient scored an 8 on the Naranjo scoring system which classifies this event as a probable drug reaction [2] (Table 1). A thorough workup for autoimmune conditions was not carried out as the patient did not have any other symptoms and has also been disease-free since her discharge. Similarly, anticoagulation workup was not performed as the patient did not have evidence of clots or thrombus on the CT scan obtained nor did she have a previous history of thromboembolism.

Data are available in the literature on various medications causing ischemic colitis with different mechanisms of action [1]. Drugs such as norepinephrine, ephedrine, phenylephrine, phentermine, methamphetamine, vasopressin, epinephrine, cocaine, ergotamine, triptans, and ephedra cause vasoconstriction in the bowel predisposing the gut to ischemia particularly at the watershed area. Antihypertensive medications such as angiotensin converting enzyme inhibitors, diuretics, calcium channel blockers, and nitrates can cause ischemia by decreasing preload and reducing the blood supply to the affected organ. Thromboembolism is implicated with the use of oral contraceptive use and hormones. Other novel mechanisms have been proposed for other medications such as vasculitis with gold salts and increase colonic luminal pressure and subsequently decreased blood supply by alosetron [3]. Several other medications are implicated with causing ischemic colitis but the mechanism of action is unclear [4].

Pseudoephedrine is a commonly used over the counter medication for cold and allergy symptoms. It is a sympathomimetic drug with a direct agonistic effect on *α*- and *β*2-adrenergic receptors causing vasoconstriction of blood vessels and relaxation of smooth muscles in the bronchi, respectively. This sympathomimetic action is likely responsible for causing ischemia in the gut as mentioned with other drugs related to same category above. After an extensive literature search, we found ten cases that suggested pseudoephedrine as the culprit agent for causing ischemic colitis [5–11] (Table 2). The dose of pseudoephedrine ingested varied across these cases with a range of 60 mg/day to 900 mg/day. Similarly, the duration of pseudoephedrine use ranged from as minimal as 5 days to 2 years. All the cases recovered uneventfully except one that required immediate surgical intervention with right-sided ischemic colitis. Our case is unique as our patient took only 3 doses of 120 mg of the drug for just one day and developed symptoms. This is the lowest possible exposure reported in the literature.

In conclusion, medications are an important factor that predisposes an individual to develop ischemic colitis. Our case report exemplifies that pseudoephedrine may cause ischemic colitis. Moreover, we would like to stress to healthcare providers (clinicians, nurse practitioners, assistant physicians, and medical students) the importance of detailed history and review of medications including prescription drugs, over the counter medications, herbal supplements, and alternative treatments to aid in the diagnosis of this condition.

## Figures and Tables

**Table 1:** Naranjo scoring algorithm for determining possibility of drug reaction, scoring: ≥ 9 = definite ADR, 5-8 = probable ADR, 1-4 = possible ADR, and 0 = doubtful ADR.

	Question	Yes	No	NA	Points
1.	Are there previous conclusive reports on this reaction? Yes (+1) No (0) NA (0)	X			1

2.	Did the adverse events appear after the suspected drug was given? Yes (+2) No (-1) NA (0)	X			2

3.	Did the adverse reaction improve when the drug was discontinued or a specific antagonist was given? Yes (+2) No (-1) NA (0)	X			2

4.	Did the adverse reaction appear when the drug was readministered? Yes (+2) No (-1) NA (0)			X	0

5.	Are there alternative causes that could have caused the reaction? Yes (-1) No (+2) NA (0)		X		2

6.	Did the reaction reappear when a placebo was given? Yes (-1) No (+1) NA (0)			X	0

7.	Was the drug detected in any body fluid in toxic concentrations? Yes (+1) No (0) NA (0)			X	0

8.	Was the reaction more severe when the dose was increased, or less severe when the dose was decreased? Yes (+1) No (0) NA (0)			X	0

9.	Did the patient have a similar reaction to the same or similar drugs in any previous exposure? Yes (+1) No (0) NA (0)			X	0

10.	Was the adverse event confirmed by any objective evidence? Yes (+1) No (0) NA (0)	X			1

**Table 2:** Previously reported dosing for pseudoephedrine in patients presenting with ischemic colitis.

S. No	Author	Year	Dose and Frequency	Sequela
1.	Schneider	1995	Pseudoephedrine 240 mg daily for 5 days	Resolved

2.	Dowd	1999	Three patients used pseudoephedrine 120 mg twice daily for 7 days and one patient used 120 mg daily for 6 months	Resolved

3.	Lichtenstein et al.	2000	Sudafed (Pseudoephedrine) 120 mg twice daily for 5 days	Resolved

4.	Klestov et al.	2001	Nucosef daily (14.9 mg codeine + 60 mg pseudoephedrine) for 2 years and 900 mg daily pseudoephedrine before each hospitalizations.	First hospitalization: Right hemicolectomy Second hospitalization: ileocolic resection

5.	Traino et al.	2004	Pseudoephedrine 240 mg per day for 7 days + tramadol 150 mg per day for chronic back pain	Resolved

6.	Sherid et al.	2014	Sudafed (Pseudoephedrine) 120 mg twice daily for 5 days	Resolved

7.	Ambesh et al.	2017	Pseudoephedrine hydrochloride, 10 mg four times daily for 1 month	Resolved

8.	Aziz et al.	2018	Pseudoephedrine 120 mg × 3 in one day	Resolved

## References

[1] Higgins P. D. R., Davis K. J., Laine L. (2004). Systematic review: the epidemiology of ischaemic colitis. *Alimentary Pharmacology & Therapeutics*.

[2] Naranjo C. A., Busto U., Sellers E. M. (1981). A method for estimating the probability of adverse drug reactions. *Clinical Pharmacology & Therapeutics*.

[3] Sherid M., Ehrenpreis E. D. (2011). Types of colitis based on histology. *Disease-a-Month*.

[4] Bielefeldt K. (2016). Ischemic Colitis as a Complication of Medication Use: An Analysis of the Federal Adverse Event Reporting System. *Digestive Diseases and Sciences*.

[5] Schneider R. P. (1995). Ischemic colitis caused by decongestant?. *Journal of Clinical Gastroenterology*.

[6] Dowd J., Bailey D., Moussa K., Nair S., Doyle R., Culpepper-Morgan J. A. (1999). Ischemic colitis associated with pseudoephedrine: Four cases. *American Journal of Gastroenterology*.

[7] Lichtenstein G. R., Yee N. S. (2000). Ischemic coliris associated with decongestant use. *Annals of Internal Medicine*.

[8] Klestov A., Kubler P., Meulet J. (2001). Recurrent ischaemic colitis associated with pseudoephedrine use. *Internal Medicine Journal*.

[9] Traino A. A., Buckley N. A., Bassett M. L. (2004). Probable ishemic colitis caused by pseudoephedrine with tramadol as a possible contributing factor. *Annals of Pharmacotherapy*.

[10] Sherid M., Samo S., Husein H., Sulaiman S., Vainder J. A. (2014). Pseudoephedrine-induced ischemic colitis: Case report and literature review. *Journal of Digestive Diseases*.

[11] Ambesh P., Siddiqui S., Obiagwu C. (2018). Pseudoephedrine Associated Ischemic Colitis. *American Journal of Therapeutics*.

